# More implicit and more explicit motor imagery tasks for exploring the mental representation of hands and feet in action

**DOI:** 10.1007/s00221-023-06718-2

**Published:** 2023-10-19

**Authors:** Federico Brusa, Mustafa Suphi Erden, Anna Sedda

**Affiliations:** 1 Psychology Department, School of Social Sciences, Heriot-Watt University, Edinburgh, UK; 2 Centre for Applied Behavioural Sciences, School of Social Sciences, Heriot-Watt University, Edinburgh, UK; 3 School of Engineering and Physical Sciences, Heriot-Watt University, Edinburgh, UK; 4 Edinburgh Centre for Robotics, Edinburgh, UK

**Keywords:** Body representation, Motor imagery, Mental imagery, Laterality task, Action monitoring

## Abstract

The mental representation of the body in action can be explored using motor imagery (MI) tasks. MI tasks can be allocated along a continuum going from more implicit to more explicit tasks, where the discriminant is the degree of action monitoring required to solve the tasks (which is the awareness of using the mental representation of our own body to monitor our motor imagery). Tasks based on laterality judgments, such as the Hand Laterality Task (HLT) and the Foot Laterality Task (FLT), provide an example of more implicit tasks (i.e., less action monitoring is required). While, an example of a more explicit task is the Mental Motor Chronometry task (MMC) for hands and feet, where individuals are asked to perform or imagine performing movements with their limbs (i.e., more action monitoring is required). In our study, we directly compared hands and feet at all these tasks for the first time, as these body districts have different physical features as well as functions. Fifty-five participants were asked to complete an online version of the HLT and FLT (more implicit measure), and an online version of the MMC task for hands and feet (more explicit measure). The mental representation of hands and feet in action differed only when the degree of action monitoring decreased (HLT ≠ FLT); we observed the presence of biomechanical constraints only for hands. Differently, when the degree of action monitoring increased hands and feet did not show any difference (MMC hands = MMC feet). Our results show the presence of a difference in the mental representation of hands and feet in action that specifically depends on the degree of action monitoring.

## Introduction

The ability to mentally recall a motor act without any overt movement is called motor imagery (MI) (Kosslyn et al. 1995). The movement simulation that occurs on a cognitive level can be seen as a way in which we express the mental representation of the body in action (Decety et al. 1994; Decety and Jeannerod 1996; Gentilucci et al. 1998; Parsons 1987; Sekiyama 1982). MI tasks can be used as a proxy for the exploration of the mental representations of the body (e.g. Brusa et al. 2021a, b; Ionta et al. 2007; Scarpina et al. 2019). Interestingly, MI tasks differ in the degree of action monitoring required to resolve the task (de Lange et al. 2008). More in detail, we can allocate MI tasks along a continuum that goes from more implicit MI tasks (less action monitoring required for the resolution of the task) to more explicit MI tasks (more action monitoring required for the resolution of the task) (Longo 2015; Scarpina et al. 2019). An example of more implicit MI tasks is provided by laterality judgment tasks (Scarpina et al. 2019), where participants are asked to judge whether the limb is a left one or a right one. Generally, hands and feet are the body parts used in such tasks (Coslett et al. 2010; Fiori et al. 2013; Ionta et al. 2007). The Hand Laterality Task (HLT) (Fiori et al. 2013) and the Foot Laterality Task (FLT) (Curtze et al. 2010) show pictures of hands and feet presented in several orientations (e.g. 0°, 90°, 180°, 270°) and views (e.g. back, palm) (Fiori et al. 2013; Funk and Brugger 2008). In the case of the HLT and FLT, the resolution of the tasks is based on a minor level of action monitoring (less awareness of the strategy used to solve the task). This means that a more implicit motor strategy is used by the participants to solve the tasks: participants judge the laterality, left or right, of limbs by mentally rotating hands and feet in their minds, without being aware of such a mental rotation (Scarpina et al. 2019). Differently, an example of more explicit MI tasks is provided by the Mental Motor Chronometry (MMC) task for hands (e.g. Brusa et al. 2021a, b; Scarpina et al. 2019) and feet (e.g. Efstathiou et al. 2022). Here, participants are openly asked to perform or to imagine performing certain movements with their hands or feet; therefore, in the case of MMC tasks, the degree of action monitoring, grounding the task resolution, highly increases, because participants are aware of using their MI skills to solve the task.

The more implicit and more explicit MI tasks do not show only differences (e.g. in the level of action monitoring) but also similarities, such as the presence of a strong relationship between physical abilities and the mental representation of the body in action (Scarpina et al. 2019). For example, in the case of laterality tasks, we can detect faster and more accurate responses to limbs when the limbs are depicted in comfortable and easy-to-reach positions (across from the body’s midsagittal plane) when compared to limbs depicted in awkward positions (away from the body’s midsagittal plane) (Brusa et al. 2021a, b; Conson et al. 2010; Parsons 1987; Scarpina et al. 2019; Sirigu et al. 1996). Similarly, for the MMC, the relationship between the physical abilities and the mental representation of the body in action is highlighted by the detection of a correlation between the time required to perform an action and the time required to mentally simulate that action (Brusa et al. 2021a, b; Scarpina et al. 2019).

Despite the importance of knowing the differences and similarities between the mental representation of hands and feet in action (e.g. for developing MI-based training (Cramer et al. 2007; Grabherr et al. 2015), or for improving the motor skills of individuals (e.g. athletes and patients) (Carrasco and Cantalapiedra 2016; Ladda et al. 2021; Milton et al. 2008)), the literature on the topic is scarce (e.g. Fiorio et al. 2006; Ionta and Blanke 2009; Ionta et al. 2007), especially when the body awareness or action monitoring (more implicit versus more explicit strategies) is involved.

Considering a peripheral physiology, hands and feet show biological similarities (e.g. the presence of five toes/fingers composed of phalanges, mid-portion, metatarsals/metacarpals, and hind portion, tarsals/carpals) (Flögel et al. 2016), but also several differences. For example, feet are characterised by a lower density of afferent and efferent fibres (Hajnal et al. 2007). Moreover, the tactile resolution of feet is lower (Weber 1846), and feet are also characterised by a higher threshold of activation for mechanoreceptors because of the greater skin thickness (Kennedy and Inglis 2002). In terms of cerebral representation of limbs, mental processes, such as MI, have been considerably explored by functional imaging methods (*for a review on the topic*: Hardwick et al. 2018; Henschke and Pakan 2023; Hétu et al. 2013). On a general level, a large fronto-parietal network is involved, as well as subcortical and cerebellar regions, when we image ourselves moving our bodies and limbs (Hardwick et al. 2018; Henschke and Pakan 2023; Hétu et al. 2013). Within this large network, upper limb MI is associated with more consistent activation in the left inferior frontal gyri and subgyral regions, while lower limb MI is associated with more consistent activation in the right supplementary motor area and left cerebellum (Hardwick et al. 2018; Hétu et al. 2013). These regions mostly overlap those areas involved in the actual motor execution of limbs (Hardwick et al. 2018; Henschke and Pakan 2023; Hétu et al. 2013).

Overall, the knowledge of the peripheral physiology and cerebral representation of upper and lower limbs tells us that hands and feet show similarities as well as differences.

Nonetheless, we do not know if the performance at the more implicit (i.e., HLT and FLT) and more explicit (MMC for hands and feet) MI tasks is influenced by peripheral physiology and cerebral representation of limbs dissimilarities, leading to different outcomes. If the latter is true, this will prove how the differences observed in peripheral physiology and cerebral representation of limbs might be reflected by specific differences in outcomes of MI tasks. To the best of our knowledge, the amount of studies that compared MI abilities for the mental representation of hands and feet in action is scarce (Fiorio et al. 2006; Ionta and Blanke 2009; Ionta et al. 2007). Moreover, these studies explored only the more implicit component of MI; the authors used the HLT and the FLT only. The results of such studies highlight the presence of biomechanical constraints for both hands and feet (Fiorio et al. 2006), and an absence of relevant differences between limbs (Fiorio et al. 2006; Ionta and Blanke 2009; Ionta et al. 2007). Only one of the studies pointed out the presence of possible differences driven by the limb factor (hand versus foot) (Ionta and Blanke 2009). More in detail, the authors observed slower Reaction times (RTs) in the recognition of feet compared to hands, across different orientations (e.g., greater RTs to recognise foot laterality for feet presented in plantar orientation, compared to a hand depicted in palm orientation) (Ionta and Blanke 2009). Further studies are then needed to better clarify the presence of possible differences in the motor mental representation of hands and feet, especially when a different degree of awareness is involved (less action monitoring versus more action monitoring).

Our study aims at exploring whether there is a difference in the MI abilities of the mental representation of hands and feet in action. Moreover, acknowledging the possibility to differentiate MI tasks because of the amount of action monitoring required, our study also aims at exploring whether more implicit and more explicit MI tasks show the same task outcomes when action monitoring is varied. For this purpose, we have launched an online study administering the HLT (Fiori et al. 2013) and the FLT (adapted from Fiori et al. 2013), as a sample of more implicit MI tasks. While, the MMC task, for hands (Brusa et al. 2021a, b; Scarpina et al. 2019) and feet (Efstathiou et al. 2022), were administered as a sample of more explicit MI tasks. Participants completed also a self-report questionnaire exploring their interoceptive body awareness (Multidimensional Assessment of Interoceptive Awareness-2; Mehling et al. 2018) (available at https://osher.ucsf.edu/maia). As the task is based on the ability to use the mental representation of the body in action, we reasoned it is important to ensure that our participants do not have extreme interoception ability. Interoception is known to be a fundamental component of body representation, influencing precision and malleability of such representation (Craig 2003; Tsakiris et al. 2011). Individuals with higher interoception (i.e. the ability to decode internal body signals) could perform worse on hand laterality tasks for example (Raimo et al. 2021); hence, this would not be representative of the population. The self-report questionnaire has been then used to screen participants after data collection to ensure none of the participants had extreme scores.

We identified the null hypothesis (Hp0) as: “the mental representation of hands and feet in action does not show any differences in its MI-related skills”. In other words, the same pattern of results would be observed independently of motor awareness, or action monitoring, required to solve the tasks. Differently, the alternative hypothesis (Hp1) we identified would lead to the following conclusion: “There are differences in the MI abilities related to the mental representation of hands and feet in action, with the possibility of observing differences in the pattern of results because of the motor awareness, or action monitoring, involved in the resolution of the tasks”.

We have hypothesised that independent of the action monitoring required, hands would show a stronger and better-defined mental representation of actions for both more implicit and more explicit tasks. This will be mirrored by better performance for hands in all of the task parameters (e.g. faster RTs, more accurate responses, stronger correlation). Our hands and feet show peripheral physiology and cerebral representation of limbs’ similarities (e.g. anatomical elements/overlap of brain regions) (Flögel et al. 2016) as well as differences (e.g. the density of afferent and efferent fibres, tactile resolution, skin thickness/specific brain regions) (Hajnal et al. 2007; Kennedy and Inglis 2002; Weber 1846). Moreover, these dissimilarities mirror the different uses of our limbs. Most of the time we use our hands to produce more fine motor actions (i.e. eye–hand coordination), while our feet are generally involved in more gross motor actions and placed out of our sight (i.e. we do not watch our feet when we are walking) (Gabbard 2008; Luo et al. 2007). In addition, feet are functionally represented in a completely different manner than the upper limb: automaticity is a feature of walking, which can be seen as almost a reflective representation (Clark 2015), whereas using the hand can even substitute speech and language (Brusa et al. 2021a, b) and is therefore used in many different ways. The peripheral physiology and cerebral representation dissimilarities between hands and feet as well as their implication on their use might be reflected by differences in the mental representation of their actions.

## Methods

The reader can download the full methods (e.g., stimuli, the syntax used to deliver the experiment, the Excel sheets for data processing, the raw and processed data, etc.) used in the current study from the OSF page: https://osf.io/bxcm5/.

### Sample

The participants were provided with a link to access the study on the online platform Psytoolkit (https://www.psytoolkit.org/) (Stoet 2010, 2017). Overall, 55 healthy individuals (mean age ± standard deviations: 24.45 years ± 5.18 years; mean education ± standard deviations: 16.67 years ± 3.30 years; 46 females) participated in the study. Participants were recruited through social media (Facebook, Twitter), word of mouth, and personal and professional contacts.

Participants who presented conditions that might influence the reliability of the collected data (e.g. neurological conditions, history of strokes and traumatic brain injury, arthritis, problems with moving hands and feet, visual problems that are not corrected with the use of glasses or lenses, and abuse of any substance that can affect thoughts, moods, and behaviour) were excluded by the study.

Both right-handed and left-handed participants were enrolled because of the absence of an a priori hypothesis on the specific role of right and left-handedness and right and left-footedness. We assessed the handedness and footedness of participants using the self-report questionnaire developed by Coren (1993). We used the data from the Coren’s (1993) self-report questionnaire to define the factor ‘limb’ as dominant or not dominant in the analyses. Our sample was composed of 9 left-handedness and 46 right-handedness participants. Differently, 7 of the participants reported left-footedness, while 48 reported right-footedness.

The interoceptive body awareness, evaluated with the Multidimensional Assessment of Interoceptive Awareness-2 (Mehling et al. 2018), experienced by participants on a group level reflected what is normally observed in the general population (i.e. scores within the 1 standard deviations cut-off). Differently, on an individual level, a few participants showed high interoceptive body awareness for one or a few more scales (i.e. scores beyond the 1 standard deviations cut-off); however, these values were generally just above the cut-off, with most of the scales within the cut-off. Therefore, we can say that all participants experienced normal interoceptive body awareness.

Written informed consent was obtained before participation. The study was designed according to the Declaration of Helsinki and received approval from the local ethical committee at Heriot-Watt University (approval number: 2020–0669-2680).

### Instrument used

#### Hand laterality task (HLT) and foot laterality task (FLT)

To explore the more implicit MI abilities of our participants in the upper and lowers limbs, participants were asked to complete the HLT and the FLT, which ask to judge the laterality of hands and feet.

For both HLT and FLT, the conceptualization of the task structure was the same (Fiori et al. 2013; Scarpina et al. 2019). More in detail, right-back/palm and left-back/palm pictures of hands (HLT) and feet (FLT) were presented in four different orientations: 0°; 90°; 180°; 270°. We used these four orientations which allow us to observe two different effects, the stimulus orientation effect and the biomechanical constraints effect (Brusa et al. 2021a, b; Conson et al. 2010; Fiori et al. 2013, 2014; Parsons 1994; Scarpina et al. 2019, 2022a, b). The stimulus orientation effect is derived by right and left limbs at 0° and right and left limbs at 180°. The stimulus orientation effect is an index indicative of visual imagery. While, the biomechanical constraints’ effect, indicating the use of MI, is computed comparing stimuli presented in an awkward position (270° left hand/90° right hand) versus stimuli presented in a comfortable position (90° left hand/270° right hand) (Brusa et al. 2021a, b; Conson et al. 2010; Fiori et al. 2013, 2014; Scarpina et al. 2019, 2022a, b). Overall, 16 pictures per limb (8 pictures of the right limb and 8 pictures of the left limb) in the back or palm perspective are used.

Each task consisted of 96 trials divided into two blocks (48 trials for each block): each stimulus was presented 6 times (3 times in the first block and 3 times in the second block) in a randomised order. To familiarize participants with the task, the two experimental blocks were preceded by one practice block, composed of six stimuli selected randomly from the full data set. Participants seat in front of the computer screen with their left and right index fingers on the “z” and “m” keys of the keyboard. They were asked to judge if the stimulus represented a right or a left limb by pressing, as quickly and as accurately as possible, the “z” key if the picture on the screen was a left limb or the “m” key if the picture was a right hand in one block, and the reverse in the other block (i.e. the “z” key to select right limb or the “m” key to select left limb) to avoid learning effects and ensure constant attention from the participant (Brusa et al. 2021a, b; Scarpina et al. 2019; 2022a, b).

A fixation cross lasting between 1000 and 1500 ms (ms) preceded each trial. The stimulus disappeared as soon as the answer key was pressed by the participant. As previously done, we left a window of 5000 ms, after which the task automatically proceeded to the next trial if the participant did not answer any key (e.g., Curtze et al. 2010). For each trial, RT (correct answers only) and the answers provided by participants were recorded. Average RT in ms and average accuracy (the percentage of correct answers) was calculated for each combination of orientation and posture.

Starting limb, hand or foot, and block order were randomised between subjects.

#### Mental motor chronometry (MMC) for hands and feet

The more explicit components of MI were explored using the MMC for hands and feet. The MMC task used in the current study was adapted for online administration (Efstathiou et al. 2022). The task is derived from Sirigu et al. (1996) and reflects the version for hands previously adopted for laboratory administration (e.g. Brusa et al. 2021a, b; Scarpina et al. 2019). It comprised two conditions named motor imagery and motor execution. As suggested by the name of the conditions, when it comes to the motor imagery condition, participants were asked to imagine a sequence of movements, differently, during the motor execution condition, participants were asked to execute the same sequence of movements. Participants were asked to imagine and execute the movements with both limbs, hands, and feet. In the case of hands, the movements were: index and thumb opposition; thumb extension from the fist; middle finger crossed on the index finger; and extension of the index and the little fingers. In the case of feet, the movements selected were: foot internal rotation; foot external rotation; foot dorsiflexion, and foot plantar flexion.

The RTs, expressed in ms, needed to imagine and execute each of the four movements were recorded. The RTs collected in the previous studies were recorded using a stopwatch (Schwoebel and Coslett 2005; Sirigu et al. 1996), differently, we used a computerised evaluation that offers a more accurate response (Armitage and Eerola 2020). Similarly to what happened for the laterality tasks, participants had the opportunity to practice the sequence of movements by a video showing each of the movements for each limb and each side. All videos were of the same length (i.e. 2 s), the movement was presented in the third person perspective, and participants could practice the movement as much as they wanted. During each experimental trial, participants were asked to read the instructions, press the spacebar, and with their eyes closed, imagine or execute the movement five times, as accurately and rapidly as possible (Sirigu et al. 1996). Participants were instructed to press the spacebar immediately after the imagery or execution of the target movement. The task was comprised of 32 trials (four movements for each limb, hand, foot, side, left and right, and condition, imagination and execution). The methods of the MMC tasks are available on the OSF page https://osf.io/6kpqx/.

### Procedure

Participants had access to the study through a link on the web platform Psytoolkit (Stoet 2010, 2017). On the first screen, participants could read the consent form and give their consent. First, the participants answered a series of screening questions that verified their eligibility for the study. Then, participants were asked to fill out a questionnaire about some demographic variables (e.g., age, gender). After that, participants completed the self-report “A questionnaire to measure hand, foot, eye and ear preference” (Coren 1993). After this first part of the general assessment, participants were invited to take a break (maximum 10 min) before starting the behavioural tasks: the more implicit MI tasks (laterality tasks: HLT and FLT) and the more explicit MI tasks (MMC for hands and feet). The order of group tasks (more implicit versus more explicit) was randomised using two different experimental links. As stated in the “Instrument used” section, participants completed the laterality task for both hands and feet. Limb and block order was randomised between subjects, in this way, we could avoid learning effects and carry-over effects. Between the more implicit and more explicit groups of tasks, a break was allowed (maximum 10 min). Concerning the MMC tasks, as stated in the “instrumented used” section, participants were asked to imagine and execute a series of movements with their eyes closed. More in detail, participants were asked to practice the movements after watching a video of each movement. This was followed by the motor imagery condition for each limb. During these trials, participants were asked to imagine performing the sequence of movements (see “Instrument used” section for target movements), with hands and feet, as quickly and as accurately as possible. After the motor imagery condition, participants completed the motor execution condition for each limb. Differently, in these trials, participants performed the movement, with their eyes closed, as quickly and as accurately as possible. All participants imagined and performed the same movements in the same order (from the index thumb opposition to the extension of the index and little fingers and from foot internal rotation to foot plantarflexion) to avoid cognitive strategies such as counting (Sharma et al. 2009). The order of the limb was randomised. The task was constituted of 32 trials.

Following the behavioural tasks, participants were asked to fill out the Multidimensional Assessment of Interoceptive Awareness-2 questionnaire (Mehling et al. 2018), to obtain a measure of interoceptive body awareness.

At the end of the experiment, a debrief screen appeared, providing participants with useful information about what happened during the study and the contact details of the authors.

The study duration was expected to be 1 h maximum, the required time depended on the duration of the breaks taken by participants, which we left flexible due to the online administration (maximum 10 min).

### Analysis pipeline

#### Power analysis

To compute the sample size necessary to observe the effect if present, a power analysis has been performed using G*Power 3.1 (Erdfelder et al. 2009; Faul et al. 2007). We carried out three different power analyses, which were related to our main analyses. The first power analysis carried out (HLT versus FLT) was based on a repeated measure analysis of variance (RM ANOVA). The project was designed as a within-subjects study, with two factors on two levels: *Limb* (Hand versus Foot) and *Posture* (Comfortable versus Awkward). The effect size observed for studies using laterality tasks is generally high (e.g. RM ANOVAs ranging from 0.13 to 0.73 η^2^, see Brusa et al. 2021a, b; Scarpina et al. 2019); however, at the time of the power analysis conducted, there were no available studies with a similar design. Therefore, we opted for a lower effect size (0.20). As for the other parameters, we have selected an a priori power of 1–− β = 0.95, with an alpha error probability of α = 0.05, one group, and four measurements as other input parameters. The output of the present power analysis resulted in a sample size of *n* = 55, providing a power of 95%.

Differently, for the second power analysis (MMC hands versus MMC feet), the main analysis was based on a correlation. That is because the isochrony component of the MMC tasks (correlation between the time required to imagine and execute the movements) is a key element of the task. The studies from the literature based on the MMC for hands revealed a strong correlation between imagined and executed movements (e.g. RM ANOVAs ranging from 0.53 to 0.70 η^2^, see Brusa et al. 2021a, b; Scarpina et al. 2019), while no data were available for lower limbs at the time of this power analysis calculation. Then, we opted for an expected correlation of *p* = 0.50 (correlation p H1). The other parameters adopted were two-tailed, an a priori power of 1–− β = 0.95, with an alpha error probability of α = 0.05, and a correlation p H0 = 0.0. The output of the present power analysis resulted in a sample size of *n* = 46, providing a power of 95%.

At last, in the third power analysis carried out, we focused our attention on a different aspect of the MMC tasks, the MMC index (Brusa et al. 2021a, b). In this case, the main analysis was an RM ANOVA with a within-subjects study of two factors, each of them on two levels: *Limb* (Hand versus Foot) and *Dominance* (Dominant versus Non-Dominant). Since this is the first time a comparison between upper and lower limbs for MMC is made, we opted for a lower effect size (0.20). We selected an a priori power of 1 − β = 0.95, with an alpha error probability of α = 0.05, one group, and four measurements as other input parameters. Similarly to the first power analysis conducted, the sample size required was *n* = 55, providing a power of 95%.

A sample size of *n* = 55 participants was chosen. The sample size chosen would ensure that results are reliable and replicable.

#### Data analysis

Data are stored in OSF: https://osf.io/bxcm5/. Data were analyzed with Statistical Package for Social Science (IBM® SPSS® Statistic, Version 26). The alpha level was set at p < 0.05 for all analyses. For HLT and FLT, for the RTs, trials in which participants gave the wrong response were discarded from the analyses. For the remaining trials, where participants gave the right answer, a cut-off of two standard deviations above and below the individual mean was used to remove outlier responses in other words’ anticipation and/or lack of attention, respectively (Ratcliff 1993; Scarpina et al. 2019).

After removing RTs’ outliers, for accuracy at the HLT and FLT, classic versions, we used a threshold of 50% accuracy for the stimuli displayed at 0° (the easiest stimuli, on which one should not expect errors) to remove responses that could indicate random guessing (Scarpina et al. 2019; Brusa et al. 2021a, b). For the HLT, the average of all four stimuli at 0° was used. In the FLT, we used stimuli displayed at 0° only as this is the most common view for feet. The totality of the responses of the participants was suppressed in such a case.

Accuracy is not a parameter of the MMC tasks; therefore, in the case of the MMC, the RTs of all responses were included; also here, we adopted a cut-off of 2 standard deviations above and below the individual mean to remove those responses indicative of anticipation and/or lack of attention, respectively (Ratcliff 1993; Scarpina et al. 2019).

Four participants were discarded, because they did not survive the data processing. Therefore, new participants were recruited to replace these four through the recruitment to ensure that the sample size of 55 was still met.

### More implicit motor imagery tasks (HLT versus FLT)

After data pre-processing, RTs and average accuracy for each orientation (0°; 90°; 180°; 270°) and perspective (palm, back), for the left and right hand separately, have been calculated.

To compare the stimulus orientation effect in hands and feet, we used a 2 by 2 RM ANOVA with *Limb* (Hand versus Foot) by *Angle of rotation* (0° versus 180°) as within-subjects factors. The same analysis has been applied to the biomechanical constraints effect, with the only difference in the factor *Posture* (Comfortable versus Awkward) instead of the factor *Angle of rotation* (0° versus 180°).

### More explicit motor imagery tasks (MMC hands versus MMC feet)

For each participant and each limb, first, we computed the average duration of each movement for the right and the left limb separately, both in the imagery and in the motor execution conditions. From these data, we defined the index for the imagery and motor execution for each side, which we called dominant and non-dominant, and for each limb, hand, and foot. As a first step, we looked for a correlation (i.e. Spearman’s correlation) between imagined and performed actions, for each limb, and separately for the dominant and non-dominant limbs (Brusa et al. 2021a, b; Scarpina et al. 2019). As for previous studies (e.g. Brusa et al. 2021a, b; Scarpina et al. 2019), in the case of a statistically significant correlation, we transformed the correlation coefficient values into z-scores (Steiger 1980). This step allows us to directly compare the correlations’ strength detected. The r-to-z transformation can be performed on the following website http://comparingcorrelations.org/ (Diedenhofen and Musch 2015).

At last, we calculated the overall MMC index (Brusa et al. 2021a, b). To compare hands and feet MMC indexes, we used a 2 by 2 RM ANOVA with *Limb* (Hand versus Foot) and *Dominance* (Dominant versus Non-Dominant) as within-subjects factors. Differently from the analyses for the more implicit MI task, here, we considered the factor dominance. In a previous cognate study with the same tasks (HLT and FLT), no differences were observed due to the dominance (Brusa, Erden, Sedda, 2021). Differently, being no studies on limb dominance for the more explicit component of MI, we decided to include here such a factor. We hypothesised a generally better performance for the dominant limb (Curtze et al. 2010; Fiorio et al. 2006; Nico et al. 2004; Takeda et al. 2010).

### Results

#### More implicit motor imagery tasks (HLT versus FLT)

#### Stimulus orientation effect

**Fig. 1:**
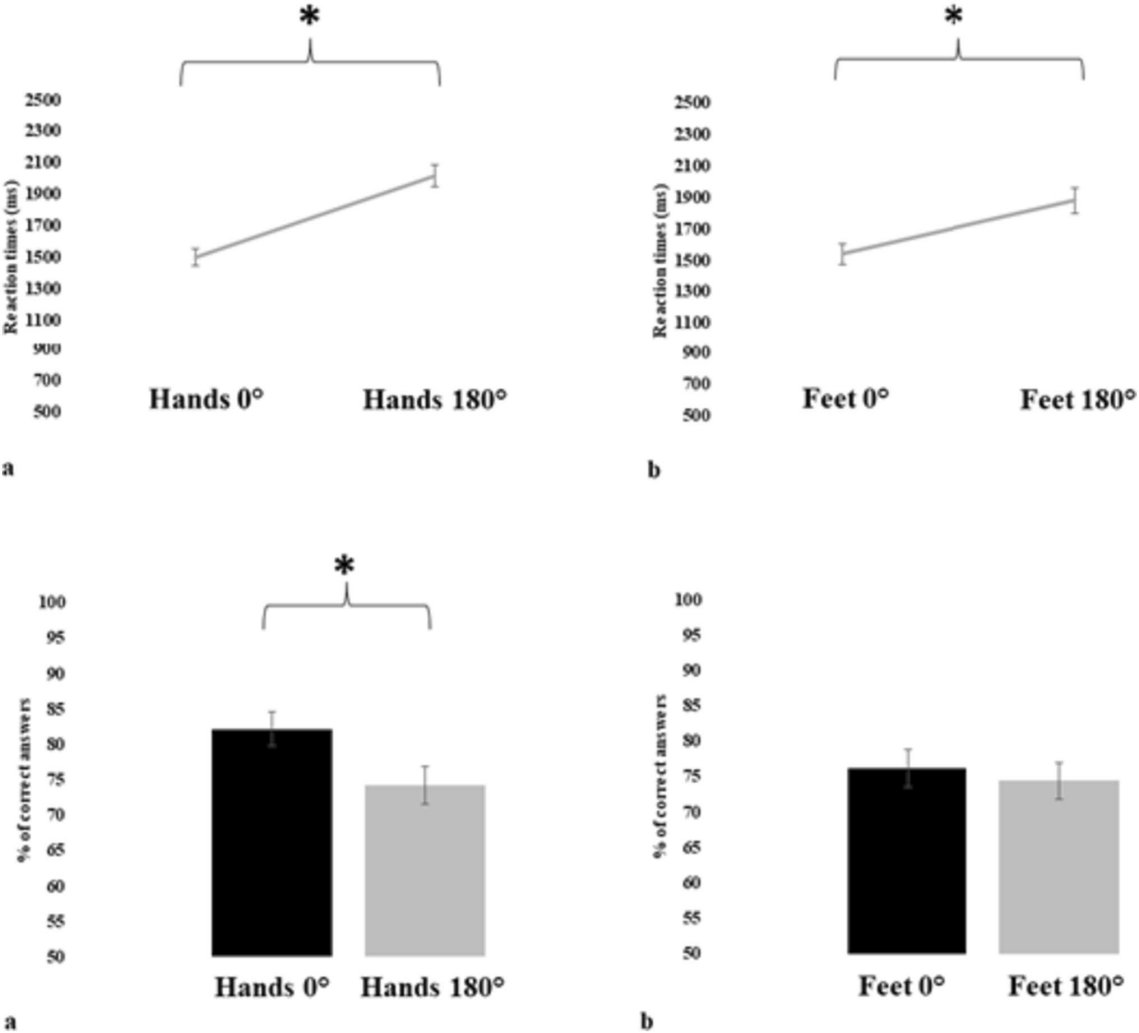
On the top of the figure, we find the graphs comparing the RTs response for stimuli presented at 0° and 180° for hands **a** and feet **b**. At the bottom of the figure we find the graphs comparing the accuracy response for stimuli presented at 0° and 180° for hands a) and feet b). The presence of significant differences is represented by lines and asterisks. Bars represent the standard error of the mean. The y-axis represents for the RTs parameter the RTs expressed in ms, and for the accuracy parameter the percentage of correct answers

We found a significant main effect of *Angle of rotation* in RTs (F(1,54) = 125.123, p < 0.001, η^2^ = 0.70), while *Limb* (F(1,54) = 0.654, *p* = 0.422, η^2^ = 0.01) main effect did not result in significative differences. As expected, participants had faster RTs with stimuli at 0° (Mea* n* = 1517 ms; ± SE = 53 ms) than with those at 180° (Mea* n* = 1948 ms; ± SE = 65 ms) independent from the *Limb*. We found a significant interaction *Limb* by *Angle of rotation* (F(1,54) = 7.375, *p* = 0.009, η^2^ = 0.12). The interaction, driven by the factor *Angle of rotation* (Fig. 1), showed faster RTs for both hands stimuli and feet stimuli at 0° (hands: Mea* n* = 1496 ms; ± SE = 55 ms; feet: Mea* n* = 1539 ms; ± SE = 66 ms) compared to the ones at 180° (hands: Mea* n* = 2018 ms; ± SE = 72 ms; feet: Mea* n* = 1879 ms; ± SE = 80 ms).

Regarding the accuracy parameter, as well as for RTs, we observed a significant main effect of *Angle of rotation* on accuracy (F(1,54) = 16.099, p < 0.001, η^2^ = 0.23), while *Limb* (F(1,54) = 2.509, *p* = 0.119, η^2^ = 0.04) main effect did not result in significative differences. Participants were more accurate with stimuli at 0° (Mea* n* = 79.2%; ± SE = 2.3%) than with those at 180° (Mea* n* = 74.3%; ± SE = 2.4%) independent from the *Limb*. The interaction *Limb* by *Angle of rotation* (F(1,54) = 5.450, *p* = 0.023, η^2^ = 0.09) was significant (Fig. 1). The interaction, driven by the factor *Angle of rotation*, showed greater accuracy of participants in response to hands stimuli shown at 0° (Mea* n* = 82.2%; ± SE = 2.5%) compared to hands stimuli at 180° (Mea* n* = 74.3%; ± SE = 2.7%). Differently, the response for stimuli picturing feet was similar between the angle of rotation 0° (Mea* n* = 76.2%; ± SE = 2.7%) and 180° (Mea* n* = 74.4% ± SE = 2.5%).

Overall, the results indicate for the RTs parameter the presence of a stable stimulus orientation effect in both hands and feet. Differently, for the accuracy parameter, the typical pattern of more accurate responses for stimuli presented with an angle of rotation of 0° than 180° was observed for hands only.

#### Biomechanical constraints’ effect

**Fig. 2:**
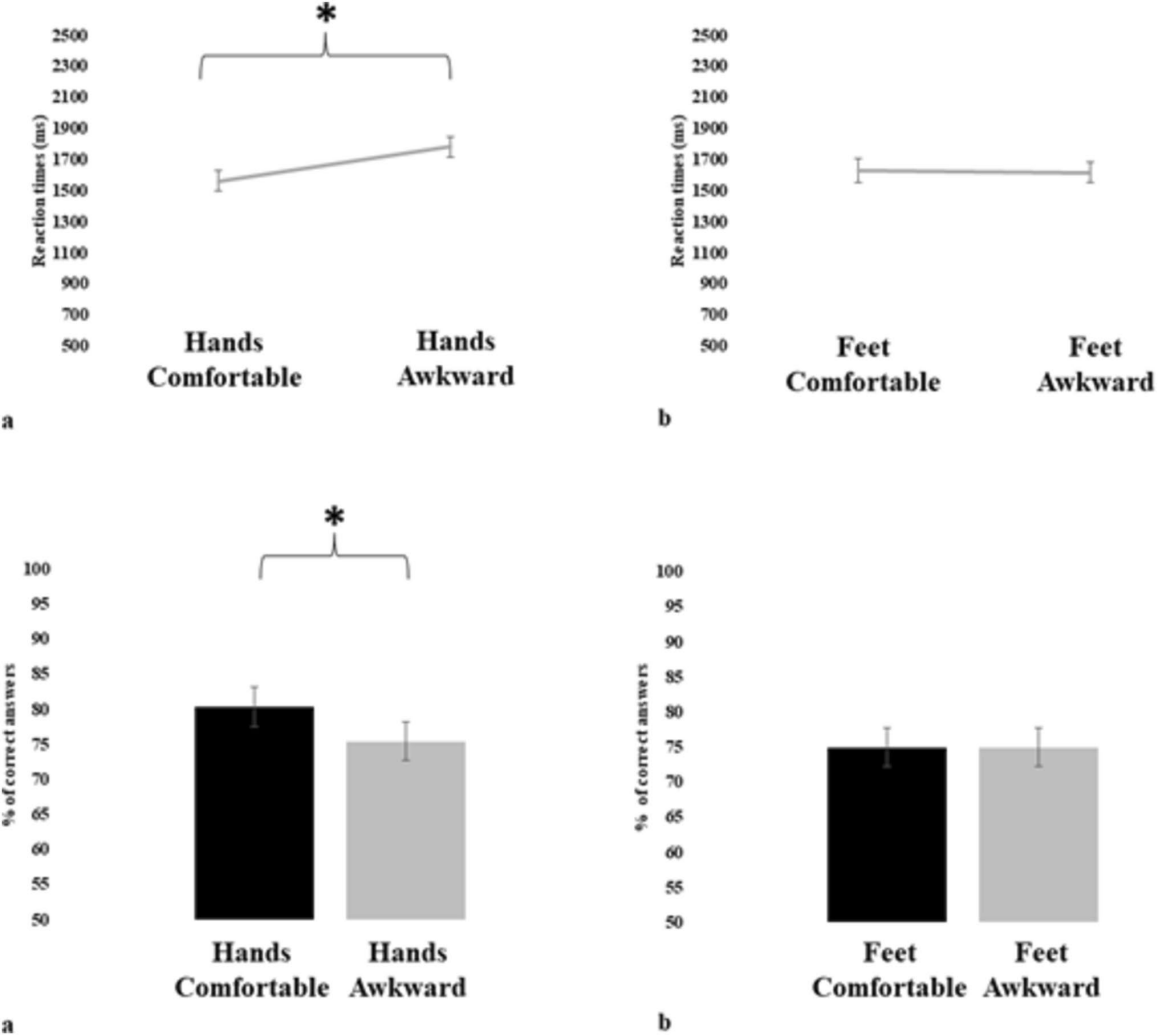
On the top of the figure, we find the graphs comparing the RTs response for stimuli presented in comfortable positions and at awkward positions for hands a) and feet b). At the bottom of the figure, we find the graphs comparing the accuracy response for stimuli presented in comfortable and awkward positions for hands a) and feet b). The presence of significant differences is represented by lines and asterisks. Bars represent the standard error of the mean. The y-axis represents for the RTs parameter the RTs expressed in ms, and for the accuracy parameter the percentage of correct answers

The RTs analysis showed a significant main effect of *Posture* (F(1,54) = 11.248, *p* = 0.001, η^2^ = 0.17): participants were faster with stimuli presented in comfortable positions (Mea* n* = 1597 ms; ± SE = 61 ms) compared to the ones presented in awkward positions (Mea* n* = 1698 ms; ± SE = 56 ms), independent from the *Limb* factor. Differently, the main effect of *Limb* (F(1,54) = 0.550, *p* = 0.461, η^2^ = 0.01) did not result in significative differences. The interaction *Limb* by *Posture* (F(1,54) = 16.513, p < 0.001, η^2^ = 0.23) was significant (Fig. 2). The interaction, driven by the factor *Posture*, showed the typical pattern of faster RTs for stimuli presented in comfortable positions (Mea* n* = 1561 ms; ± SE = 66 ms) compared to the ones presented in awkward positions (Mea* n* = 1781 ms; ± SE = 63 ms) only for hands. The RTs collected for stimuli picturing feet were similar between comfortable (Mea* n* = 1632 ms; ± SE = 78 ms) and awkward (Mea* n* = 1615 ms ± SE = 66 ms) positions.

Similarly to RTs, we found a significant main effect of *Posture* (F(1,54) = 5.832, *p* = 0.019, η^2^ = 0.09), while *Limb* (F(1,54) = 2.262, *p* = 0.138, η^2^ = 0.04) main effect did not result in significative differences. Participants were more accurate with stimuli shown in comfortable positions (Mea* n* = 77.7%; ± SE = 2.6%) than with those shown in awkward positions (Mea* n* = 75.2%; ± SE = 2.5%), independent from the *Limb*. The interaction *Limb* by *Posture* (F(1,54) = 4.114, *p* = 0.047, η^2^ = 0.07) was significant (Fig. 2). The interaction, driven by the factor *Posture*, showed, as for RTs, greater accuracy of participants in response to hands stimuli presented in comfortable positions (Mea* n* = 80.3%; ± SE = 2.9%) compared to hands stimuli presented in awkward ones (Mea* n* = 75.5%; ± SE = 2.7%). This pattern was not present for feet; the accuracy observed was similar between comfortable (Mea* n* = 75.0%; ± SE = 2.8%) and awkward (Mea* n* = 75.0% ± SE = 2.7%) positions.

The results indicate the presence of biomechanical constraints effects in the hands only. For feet, the typical pattern of better responses, faster RTs, and greater accuracy was not observed for stimuli presented in comfortable positions, compared to the ones presented in awkward positions.

Overall, when the mental representation of hands and feet in action is compared using more implicit MI measures, we observed a specific difference: feet do not show the biomechanical constraints effect.

#### More explicit motor imagery tasks (MMC hands versus MMC feet)

**Fig. 3:**
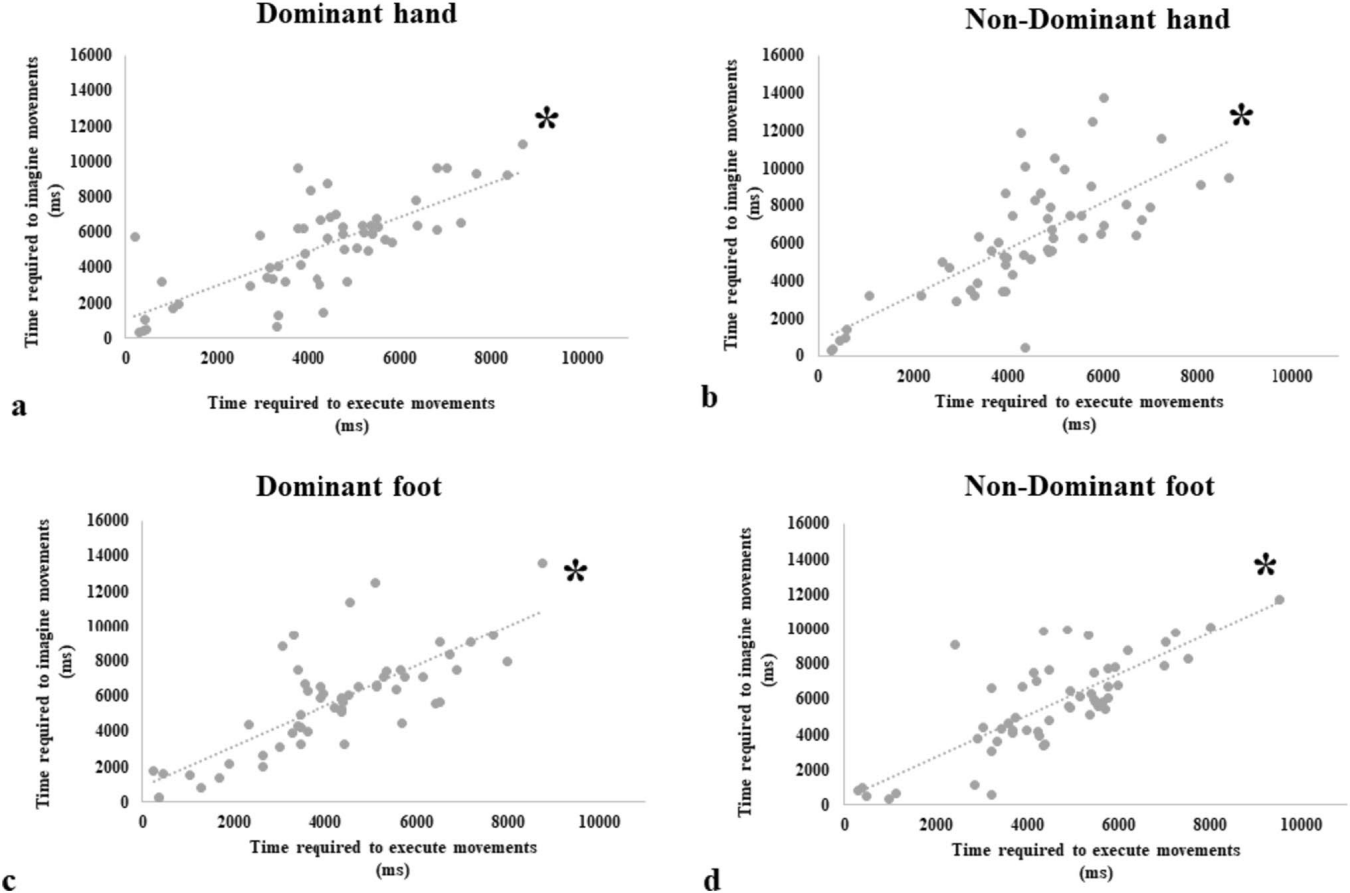
Isochrony (correlation between execution and imagery) for the MMC task. Top row: data for the dominant (**a**) and non-dominant (**b**) hand. Bottom row: data for dominant (**c**) and non-dominant (**d**) foot. Asterisk indicates a significant correlation

As expected, participants showed a significant, positive, correlation between the time required to imagine and the time required to execute movements for the dominant hand (*p*(53) = 0.701; *p* < 0.001; two-tailed), non-dominant hand (*p*(53) = 0.769; *p* < 0.001; two-tailed), dominant foot (*p*(53) = 0.741; *p* < 0.001; two-tailed) and non-dominant foot (*p*(53) = 0.720; *p* < 0.001; two-tailed) (Fig. 3).

Then, we compared the correlations’ strength transforming the correlation coefficient values into z-scores (Steiger 1980). The comparison between the strength of the correlation coefficients in the dominant hand versus the dominant foot did not identify any difference [*z* =  − 0.54; *p* = 0.58]. The same pattern occurred for the comparison between non-dominant hands versus non-dominant foot [*z* = 0.74; *p* = 0.46]. Since the absence of significant results, we can conclude that for both dominant and non-dominant limbs the relationship between the time required to execute the movements and the time required to imagine them is similar.

The analysis of the MMC index showed an absence of significant differences for the main factor *Limb* (F(1,53) = 0.130, *p* = 0.720, η^2^ = 0.002). Differently, the main factor *Dominance* resulted in significant differences (F(1,53) = 4.451, *p* = 0.040, η^2^ = 0.08); participants were faster in the imagination and execution of the movements with the dominant limb (Mea* n* = 4835 ms; ± SE = 299 ms) compared to the non-dominant one (Mea* n* = 5118 ms; ± SE = 292 ms), independent from the *Limb* factor. The interaction *Limb* by *Dominance* was not significant (F(1,53) = 3.270, *p* = 0.076, η^2^ = 0.06). Results suggest that the MMC index of hands and feet does not differ.

Overall, results suggest that the mental representation of hands and feet in action, as assessed by a more explicit MI measure, does not differ.

### Discussion

The mental representation of the body in action can be explored through MI tasks, which are allocated along a continuum, going from more implicit tasks (e.g. HLT and FLT) to more explicit tasks (e.g. MMC tasks for hands and feet) (Longo 2015; Scarpina et al. 2019). Moreover, the more implicit tasks (i.e. HLT and FLT) trigger MI using external stimuli (e.g. the picture of a right hand), while in the more explicit tasks (i.e. MMC for hands and feet), the individuals need to internally generate the imagery. The literature lacks direct comparisons between MI abilities of hands and feet, especially when the weight of body awareness, or action monitoring strategies, is taken into account. With the current study, we aimed at exploring whether the MI abilities differ between hands and feet and whether the same pattern of results occurs within more implicit and more explicit MI tasks. We compared the performances of participants at more implicit MI tasks, such as the HLT and the FLT, and more explicit MI tasks, such as the MMC for hands and feet.

It was hypothesised that more implicit and more explicit MI tasks would both show faster RTs, more accurate responses, and a stronger correlation in all the task parameters for hands, if independent of the action monitoring required hands showed a stronger and more defined mental representation of actions. Our results partially confirm the hypothesis; the data show a difference: when the degree of action monitoring decreases, the mental representation of hands and feet in action differs, while it remains similar when the degree of action monitoring increases.

Let us look first at the more implicit MI tasks, the HLT and the FLT. In terms of stimulus orientation effect, the visual imagery component of the tasks, hands and feet differ only in the accuracy parameter. When the stimuli are feet, the accuracy is similar between the two angles of rotation (0° = 180°). Differently, hands show better performance (more accurate responses) for stimuli at 0° compared to the ones at 180°, as expected. The discrepancy between RTs and accuracy for feet might be explained by the amount of exposure to the limbs. We are less often exposed to our bare feet, which most of the time are covered with socks and shoes, especially if you live in cold places such as Scotland. Differently, daily, we follow our bare hand movements for eye-hand coordination (Battaglia-Mayer and Caminiti 2018). The different visual exposure to our limbs might have led to the differences observed in the accuracy parameter, as though seeing our feet less reduces our accuracy in their visual recognition. However, the reason why participants were less accurate in differentiating feet, but not hands could also be related to the visual features discriminating between feet and hands. The toes of a foot do not create a shape where the thumb becomes an anchor point (as occurs for hands) which helps in recognising the limb, hand, or foot, as such (Conson et al. 2021).

Considering the biomechanical constraints effect, the MI index, feet do not show the presence of biomechanical constraints in RTs or accuracy, while hands show the presence of a biomechanical constraints effect in both parameters. It is known from the literature (Conson et al. 2010; Parsons 1994; Scarpina et al. 2022a, b; Sekiyama 1982) that when the typical pattern of the biomechanical constraints effect does not occur, this might indicate the use of a more general visual imagery strategy in solving the task. Thus, foot stimuli might have been treated as objects rather than as body parts in the resolution of the FLT. However, it is also possible that the FLT, when compared to the HLT, is less sensitive as a more implicit measure to capture the mental representation of feet in action. Only a few studies used the FLT and compared directly HLT and FLT; therefore, it might be possible that results for feet are less robust with these types of tasks when compared to the ones for hands, where results are more consistent. From a methodological perspective, someone might point out that the absence of the stimulus orientation effect for accuracy and the general absence of the biomechanical constraints effect for feet stimuli might be due to a compatibility effect between the stimulus depicted and the mode of response. Participants responded to both hands and feet stimuli by pressing a key on a keyboard. However, as previously reported, the previous studies that used the FLT recorded the answers verbally (Fiorio et al. 2006; Ionta and Blanke 2009; Ionta et al. 2007) or with a keyboard (Curtze et al. 2010). This did not influence the observation of biomechanical constraints. As such, it is not possible to assume that the absence of the effect of the biomechanical constraint is due to a compatibility effect; rather, the use of a more general visual imagery strategy in solving the task (e.g. foot stimuli treated as objects rather than as body parts) and the lower sensibility of FLT as a more implicit measure to capture the mental representation of feet in action is a more convincing explanation.

Moving on to the results within the more explicit MI tasks, the MMC task for hands and feet, the main picture changes. In terms of isochrony, both hands and feet are characterised by a positive correlation between the time required to imagine a movement and the time required to execute it. However, this first analysis did not exclude the presence of a difference in the strength of the correlations. Thus, we have compared the strength of the correlations between the dominant hand and foot and the non-dominant hand and foot. The strength of the correlations between the dominant hand and dominant foot was the same as for the non-dominant ones. Moreover, the evaluation of the general MMC index of hands and feet did not reveal significant differences; in fact, the data highlighted a general effect of dominance. For the dominant limbs, the time required to imagine and execute movements is shorter (< RTs). The presence of a dominant effect, in which dominant limbs are faster in movement imagination and execution, is in line with the studies of single and bimanual task and limb dominance (e.g. Shen and Franz 2005). Overall, the ability to imagine and execute movements did not differ between hands and feet; no significant difference was observed for the more explicit MI measure.

Taken together, the results show a difference between the mental representation of the body in action for hands and feet only for the more implicit measures of MI. In terms of more explicit MI measures, hands and feet are similar. As mentioned above, the two categories of tasks differ in terms of action monitoring required by the participants to solve the tasks (de Lange et al. 2008). The resolution of the HLT and the FLT is based on more implicit mental rotation strategies that participants are not aware of using (e.g. reduced action monitoring). In the MMC tasks, participants are explicitly asked to imagine and execute movements (i.e. increased action monitoring). Moreover, in the laterality tasks, an external stimulus (e.g. the picture of a right hand) triggers MI, while in the MMC tasks, individuals need to internally generate the imagery of actions. Thus, since action monitoring is more implicit in laterality tasks and the MI is triggered automatically by the stimulus, this might facilitate the expression of differences in the mental representation of hands and feet action (i.e. you are less aware of the use of your body to solve the task). However, in the MMC tasks, the differences in the mental representation of upper and lower limbs in action might be less detectable, because the imagery of actions is generated by free will, as though the possibility of actively monitoring actions erased the differences in the mental representation of hands and feet (i.e. you are more aware of the use of your body to solve the task). If this is the case, to explore the differences between the mental representation of hands and feet in action, it would be better to use paradigms based on reduced action monitoring (e.g. laterality-based), which would allow the expression of such differences.

Another difference between laterality tasks and MMC tasks is related to the types of movements involved in the tasks. The MMC for feet is based on more gross motor actions (Bakker et al. 2008; Cramer et al. 2007, 2018; Hotz-Boendermaker et al. 2008; Jackson et al. 2003, 2004; Malouin et al. 2007), while the MMC task for hands is based on finer motor actions (Brusa et al. 2021a, b; Schwoebel and Coslett 2005; Sirigu et al. 1991). For this reason, the absence of differences between the mental representation of hands and feet in action in this type of task might be related to the fact that the tasks assessed the MI abilities associated with the actual motor skills of the limbs (i.e. we are more used to performing gross motor actions with feet and fine motor actions with hands (Gabbard 2008; Luo et al. 2007)). In contrast, in the FLT and the HLT, this discrimination between grosser and finer motor actions is not so obvious. This could be the reason why only in the case of more implicit MI tasks have we observed a difference in the mental representation of hands and feet in action.

At last, because of the more implicit nature grounding the resolution of the laterality tasks (if compared to the MMC ones) (Brusa et al. 2021a, b; Scarpina et al. 2019), it is assumed in the study that participants were unaware of using MI, as they were not told to use it. However, we cannot exclude the presence of individual differences (e.g. more awareness on own MI abilities (McAvinue and Robertson 2008), and greater motor expertise (Habacha et al. 2014) that might have led the participants to use their MI skills in the resolution of the HLT and FLT despite the absence of an explicit request in relying on MI to solve the tasks. We were not able to control for this. Upcoming studies that will compare more implicit and more explicit MI tasks might consider the role of individual differences asking participants what strategy they have implied to solve the tasks; this would tell us if MI has been adopted consciously or not by participants when it comes to more implicit MI tasks. Similarly, the next studies might introduce a Likert scale to evaluate the awareness of using MI skills when solving the tasks (e.g. on a scale from 0 to 10) at the end of each task. This would give us a quantifiable self-measure of the awareness used in the use of MI even if participants were not instructed to rely on it for solving the tasks.

It should be noted that palm and back difference is an important feature in the HLT involving hands (e.g. Conson et al. 2021). This is the reason why we did include stimuli from both perspectives in our study. However, we are also conscious that our study aims at exploring differences between hands and feet, focusing on the level of awareness required from the task. The MMC task does not have the feature perspective. Given that the perspective analysis would then be relevant only for one of our tasks, and would not contribute much to the overall aim of the study, we decided not to include this analysis.

Despite the behavioural nature of the study, it is important to consider hands and feet different cerebral representations to fully understand the implications of our study. As mentioned in the introduction, when we imagine ourselves moving our bodies, a fronto-parietal network, as well as subcortical and cerebellar regions, are activated (Hardwick et al. 2018; Henschke and Pakan 2023; Hétu et al. 2013). However, imaging moving a hand (i.e. upper limb) or a foot (i.e. lower limb) changes the consistency of activation within this general MI network (Hardwick et al. 2018; Hétu et al. 2013). In addition, the nature of the MI tasks, more implicit or more explicit, influences the activation of specific brain areas with consistent activations of the right hemisphere for more implicit tasks and consistent bilateral activations for more explicit MI tasks (Hétu et al. 2013). This latter finding concerns upper limbs only, no data are available on this for lower limbs (Hétu et al. 2013). Hence, the missing piece of the puzzle is to use a protocol similar to ours in terms of behavioural tasks, with different degrees of awareness required and different body parts involved, complementing it with a neuroimaging measure to capture cerebral activation data for lower limbs.

Overall, the mental representation of hands and feet in action differed only when the degree of action monitoring decreased (HLT ≠ FLT); we observed the presence of biomechanical constraints only for hands. However, when the degree of action monitoring increased, hands and feet did not show any difference (MMC hands = MMC feet).

Our results, paralleling similar findings in peripersonal space (Gherri et al. 2022), show a difference in the mental representation of hands and feet in action specific to the degree of action monitoring considered.

## Data Availability

The data and materials of the experiment are available on the OSF page: https://osf.io/bxcm5/.
